# Effect of resveratrol on the antioxidant enzyme pathway and alveolar bone loss in experimental periodontitis

**DOI:** 10.2340/aos.v84.44956

**Published:** 2025-12-15

**Authors:** Yasemin Sezgin, Mehtap Bilgin Çetin, Yunus Kasim Terzi, Özlem Darcansoy İşeri, Hilal Erinanç, Feride İffet Şahin, Batuhan Bakırarar, İdil Özerkliğ, Şule Bulut, Nilgün Özlem Alptekin

**Affiliations:** a Department of Periodontology, Faculty of Dentistry, Baskent University, Ankara, Turkey; b Department of Dental Hygiene, College of Health Sciences, University of Doha for Science and Technology, Doha, Qatar; c Department of Medical Genetics, Faculty of Medicine, Baskent University, Ankara, Turkey; d Department of Molecular Biology and Genetics, Faculty of Science and Letters, Baskent University, Ankara, Turkey; e Department of Pathology, Faculty of Medicine, Baskent University, Konya, Turkey; f Process Management Coordinator/Biostatistician, ADO Group, Antalya, Turkey

**Keywords:** Animal model, antioxidant enzymes, oxidative stress, periodontitis, resveratrol

## Abstract

**Objective:**

The purpose of this study was to investigate the effect of resveratrol in a rat model of experimental periodontitis by assessing alveolar bone loss along with catalase and glutathione peroxidase mRNA expression and enzymatic activity in gingival tissue.

**Material and methods:**

Experimental periodontitis was induced in 34 male Sprague-Dawley rats by placing silk ligatures bilaterally around mandibular first molars. Then the animals were randomly divided into two groups: placebo (*n* = 17) and resveratrol (*n* = 17). The placebo group received a placebo solution, while the resveratrol group received 10 mg/kg resveratrol daily via oral gavage for 30 days. Alveolar bone loss was measured on microscopic photographs of stained tissue sections. Gingival enzyme activity levels were determined by enzyme-specific reactions and mRNA levels were assessed using semiquantitative real-time polymerase chain reaction (PCR).

**Results:**

Systemic resveratrol administration significantly reduced alveolar bone loss (440.87 ± 142.24 μm) compared to the placebo group (897.06 ± 383.59 μm) (*p* < 0.001). Catalase and glutathione peroxidase activities were also significantly higher in the resveratrol group (*p* < 0.001).

**Conclusions:**

Resveratrol appears to be effective in reducing alveolar bone loss in experimental periodontitis, potentially through its antioxidant properties. These findings suggest that resveratrol may offer clinical benefit as an adjunct to periodontal therapy.

## Introduction

Periodontitis is characterized by the progressive destruction of the supporting periodontal tissues. The progression of periodontitis is considered a result of the complex relationship between pathogens in the plaque biofilms and the host immune response [1, 2]. Globally, periodontitis affects nearly 45–62% of adults, while severe forms are present in about 10–24% of the population [3, 4].

Beyond its local destructive effects, periodontitis has been linked to several systemic conditions, including diabetes mellitus, cardiovascular disease, respiratory diseases, and adverse pregnancy outcomes, highlighting its significant public health and clinical relevance [5–7].

Systemic inflammatory response is accompanied by oxidative stress, which plays a major role in initiating the disease. Inflammatory cells produce pro-inflammatory cytokines that induce reactive oxygen species (ROS), ultimately leading to the destruction of periodontal tissues and alveolar bone loss [1, 8]. To counteract this damage, cells rely on enzymatic and non-enzymatic antioxidant defense systems. Among them, enzymatic antioxidants are the first line of defense against ROS and described as preventative and repressive compounds[9, 10]. Thus, enhancing the host’s antioxidant capacity by using exogenous antioxidants might be critical in preventing periodontal tissue destruction [11].

Resveratrol (RESV, 3, 4’, 5-trihydroxystilbene) is a polyphenolic compound that has antioxidant, anti-cancer, antibacterial, antiviral, anti-inflammatory, anti-aging, antifungal, and antithrombotic activity and has beneficial effects on diabetes, cardiovascular diseases, neurodegenerative diseases, and modulating bone metabolism [12–15].

Previous studies have demonstrated that resveratrol can inhibit osteoclast differentiation and activity, thereby reducing bone resorption. In addition, several experimental studies suggest that resveratrol may promote osteoblast differentiation and bone formation. Through these dual mechanisms, resveratrol contributes to the preservation of alveolar bone in inflammatory conditions such as periodontitis [16–20].

With regards to antioxidant activity, resveratrol has been shown to be a scavenger of free radicals, but the direct scavenging activities of resveratrol are relatively poor. Previous studies showed that resveratrol induces antioxidant activities by activating AMP-activated protein kinase (AMPK) via sirtuin 1 (Sirt 1) and nuclear factor E2-related factor 2 (Nrf2)/ antioxidant defense pathway [21, 22]. Resveratrol has also been shown to enhance the effect of catalase (CAT) and glutathione peroxidase (GPx) antioxidant enzymes [23, 24].

To date, some studies have investigated the protective effects of resveratrol on alveolar bone loss in experimental periodontitis models [12, 21, 25–30]. Among these, a limited number of studies have evaluated its impact on antioxidant enzymes. In rat experimental periodontitis models, two studies have reported that resveratrol increased superoxide dismutase-1 activity in gingival tissues in diabetic [31] and smoke-inhalation [32] models. Yet none of these studies have evaluated the effect of resveratrol on CAT and GPx enzyme activities or gene expression levels in gingival tissue.

Based on this evidence, the present study was conducted to determine the effect of resveratrol on periodontal tissue damage in a rat experimental periodontitis model by evaluating alveolar bone loss and *Catalase (E.C: 1.11.1.6)* and *GPx (E.C: 1.11.1.9)* mRNA levels and activities in gingival tissue.

## Materials and methods

### Animals

Thirty-four male Sprague-Dawley rats weighing 300–400 g (10 weeks of age) were obtained from the Department of Medical Science Application and Research Centre of Baskent University. The rats were acclimatized for 15 days before the study; they were housed in temperature-controlled individual cages, exposed to a 24-h light-dark cycle of equal time, and had access to water and food *ad libitum*. All animal experiments were performed between January and February 2017. Baskent University Animal Ethics Committee approved all the experimental procedures (Project no D-DA16/01). In addition, the Guidelines for Animal Research: Reporting of In Vivo Experiments (ARRIVE) were included.

## Treatment groups

**Figure 1:**
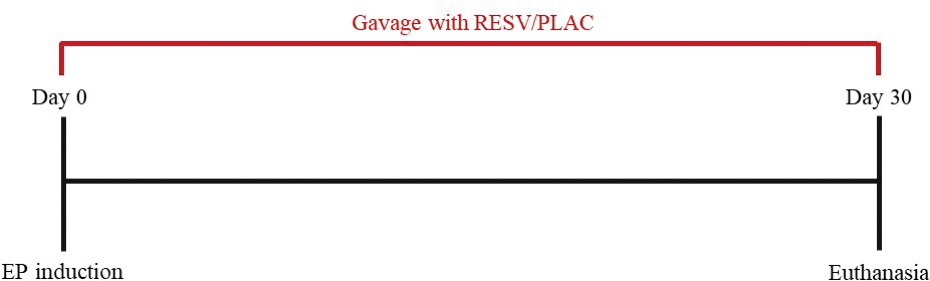
Schematic illustration of the experimental design. EP: experimental periodontitis, RESV: resveratrol, PLAC: placebo.

The experimental design is illustrated in Figure 1. The animals were randomly assigned to one of two groups by a blinded researcher via closed envelope system: (1) placebo group (PLAC) (*n* = 17), which received daily administration of a placebo solution, and (2) resveratrol group (RESV) (*n* = 17), which received daily administration of 10 mg/kg resveratrol via gavage for 30 days [14, 21, 33]. A stock solution of RESV (R5,010–500MG, Sigma-Aldrich, Sao Paulo, Brazil) was prepared in dimethyl sulfoxide and further diluted in water for working concentrations. The placebo solution was composed of the exact quantities of dimethyl sulfoxide and water in the preparation of RESV.

### Rat periodontitis model

To induce experimental periodontitis, bilateral mandibular first molars received a silk ligature (3–0) (Dogsan Ilac Sanayi, Istanbul, Turkey) in a cervical position knotted subgingivally, at the cemento-enamel junction[34, 35]. Maxillary first molars were left unligated. Ligatures were kept in position for 30 days. This procedure was performed under general anesthesia by intra-peritoneal administration of ketamine (50 mg/kg) and 2% xylazine (8 mg/kg) by a blinded researcher. The animals were evaluated daily to control possible clinical or toxicologic symptoms and to check the ligatures. On day 30, the animals were euthanized using cardiac puncture under intraperitoneal anesthesia with ketamine hydrochloride (35 mg/kg) and xylazine (3 mg/kg). Mandibles and maxillae were excised for histometric analysis. The buccal gingival tissues surrounding the mandibular first molars were collected, quickly frozen in liquid nitrogen, and stored at ‐80°C for subsequent molecular and biochemical analysis.

### Measurement of alveolar bone loss

The maxillae and mandibles from all experimental groups were fixed in 10% buffered formalin and decalcified in 10% formic acid for 24 h [36]. Following decalcification, samples were embedded in paraffin wax. Serial mesiodistal sections, 5 μm thick, were prepared using an automated tissue processing machine (Leica ASP 300, Microsystems, Germany) [37, 38]. After processing, sections were embedded in paraffin, and routine histological sections of 3–4 μm thickness were obtained using a conventional microtome and stained with hematoxylin and eosin (H&E) for microscopic examination.

Measurements were conducted using a digital camera system (Olympus UTV1XC, Tokyo, Japan) connected to the microscope at 20× magnification. Microscopic images were captured and analyzed using ImageJ software (National Institutes of Health, USA). The linear distance (μm) between the cementoenamel junction (CEJ) and the top of the alveolar bone crest (ABC) was measured [37, 38].

**Figure 2:**
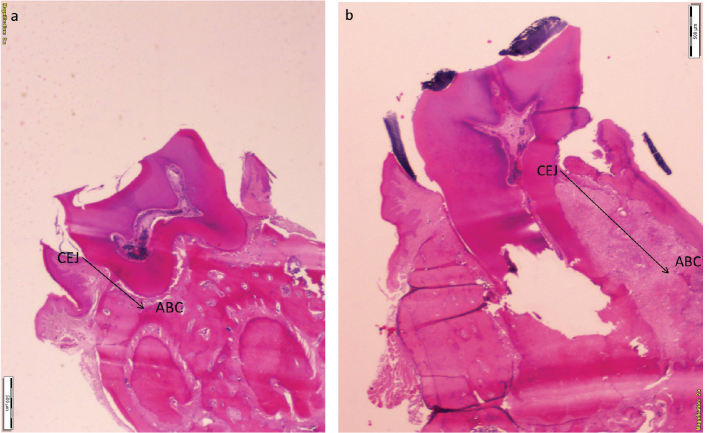
Photomicrographs of the stained tissue sections showing (A) the ligated-resveratrol group and (B) the ligated-placebo group. A reference line indicates the distance between the cemento-enamel junction and the alveolar bone crest, illustrating alveolar bone loss. (hematoxylin and eosin; original magnification x20).

As per established protocols, five serial mesiodistal histological sections were analyzed, and the mean CEJ–ABC linear distance for each tooth was calculated (Figure 2). Measurements were performed by a blinded examiner following intra-examiner calibration. Calibration was achieved through repeated measurements of five representative histological images with alveolar bone loss patterns similar to the current study. Measurements were performed twice within a 24-h period to assess repeatability. The intra-class correlation coefficient (ICC) demonstrated 95% reproducibility.

### Expression analysis of CAT and GPx1 genes

Expression analysis of the CAT and GPx1 genes was performed on gingival tissue samples using reverse transcription polymerase chain reaction. (RT-qPCR). Total RNA was isolated with TriPure isolation reagent according to the manufacturer’s instructions (Roche Diagnostics GmbH, Mannheim, Germany). The quality and quantity of RNA were determined by using NanoDrop 2000 Spectrophotometer (Thermo Scientific, Wilmington, DE, USA). One microgram of total RNA was reverse transcribed using iScript cDNA Synthesis Kit (Bio-Rad Laboratories, Inc., Hercules, CA, USA).

Expression levels of CAT and GPx1 were determined via RT-qPCR. Primer sequences for CAT, GPx1, and beta-actin (ACTB) are given in Table 1. RT-qPCR was carried out using iTaq Universal SYBR Green Supermix (Bio-Rad Laboratories, Inc., Hercules, CA, USA). PCR reactions were performed with the CFX96 Touch Real-Time PCR Detection System (Bio-Rad), briefly: after first denaturation at 95°C for 1 min, 40 cycles; 95°C for 10 s, 60°C for 30 s. Beta-actin (*ACTB*) was used for the normalization of expression data. Relative changes in gene expression levels were calculated using the 2^-∆∆CT^ method [39].

**Table 1:** Sequences, amplicon sizes, and melting temperatures of the primers used in RT-qPCR.

Gene	Forward primer (5’-3’)	Reverse primer (5’-3’)	Amplicon size (bps)	Tm (°C)
*CAT*	ACAACTCCCAGAAGCCTAAGAATG	GCTTTTCCCTTGGCAGCTATG	76	60
*GPx1*	GTGCAATCAGTTCGGACATCA	CACCGGGTCGGACATACTTG	77	60
*ACTB*	GGGAAATCGTGCGTGACATT	GCGGCAGTGGCCATCTC	76	60

bps: base pairs, Tm: melting temperature.

### Analysis of catalase (E.C: 1.11.1.6) and glutathione peroxidase (E.C: 1.11.1.9) activities

Approximately 50 mg of mandibular gingival tissue, obtained by pooling samples of 2–3 rats, was homogenized in 1 mL ice-cold homogenization buffer (1.15% (w/v) KCl, 5mM EDTA, 0.2mM PMSF, 0.2mM DTT in 25 mM phosphate buffer, pH 7.4) using a glass-glass homogenizer. The homogenates were centrifuged at 1.500g, and the supernatants were sampled to determine enzyme activities. The total protein amount in supernatants was determined according to the Bradford method [40].

For the determination of catalase activity [41], cytoplasmic fractions were diluted five times in 1% (v/v) Triton X‐100. The reaction was initiated by adding H_2_O_2_ to phosphate buffer (pH 7), and the decrease in the absorbance of H_2_O_2_ was recorded at 240 nm for 1 min against assay medium (ε = 39.4 mM^-1^ cm^-1^). One unit of catalase activity was defined as the amount of substrate (μmol) consumed in one min by 1 mg total protein.

GPx activity was measured based on the nicotinamide adenine dinucleotide phosphate (NADPH) oxidation with glutathione reductase, which uses oxidized glutathione and NADPH as substrates [42]. Since oxidized glutathione is produced by GPx, the degree of NADPH oxidation is directly proportional to GPx activity. The assay medium contained reduced glutathione (GSH), glutathione reductase, 40-fold diluted cytosolic fraction, and NADPH in Tris buffer (pH 8). The enzymatic reaction was initiated with the addition of H_2_O_2_, and the rate of decrease in NADPH was measured spectrophotometrically at 340nm (ε 340 = 6220 M^‐1^.cm^‐1^) for 3 min. GPx activity was described as the amount of NADPH consumed in 1 min by 1 mg protein containing cytosolic fraction.

### Sample size

According to the power analysis, a minimum of 17 animals per group were required to detect statistically significant differences (*a* = 0.05) with 90% power [43].

### Statistical analysis

The data were analyzed using SPSS version 11.5. Descriptive statistics were presented as mean ± standard deviation and median (minimum–maximum) for quantitative variables, and as number (percentage) for qualitative variables. To compare the categories of a qualitative variable with two groups in terms of a quantitative variable, the Student’s *t*-test was used when the assumptions of normal distribution were met; otherwise, the Mann–Whitney *U* test was applied. For comparisons between two dependent quantitative variables, the Paired *t*-test was used when the assumptions of normal distribution were satisfied, and the Wilcoxon Signed Rank test was applied otherwise. A *p* < 0.05 was considered statistically significant.

## Results

### Histometric results

**Figure 3:**
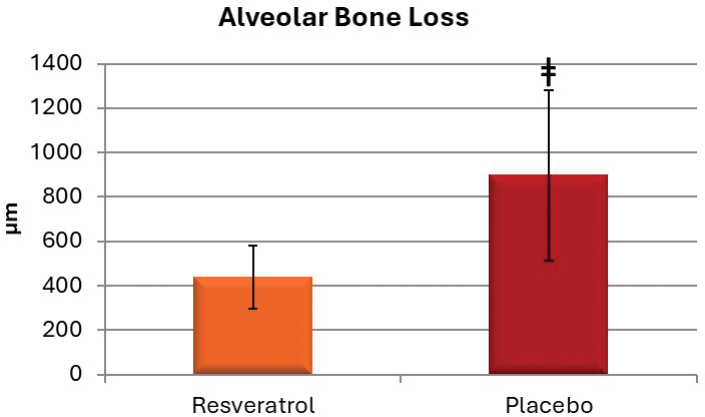
Mean ± SD of alveolar bone loss (micrometers) for resveratrol and placebo groups. ǂRepresents significant difference between groups (Mann-Whitney *U* test; *p* < 0.05).

Mean alveolar bone loss in the ligated-PLAC group was 897.06 ± 383.59 μm, while the unligated-PLAC showed significantly lower bone loss at 306.35 ± 83.12 μm. A significant difference in alveolar bone loss was observed between ligated and unligated teeth in the PLAC (*p* < 0.001). Mean alveolar bone loss in the RESV group (440.87 ± 142.24 μm) was significantly less compared to the PLAC (897.06 ± 383.59 μm) (*p* < 0.001) (Figures 2 and 3).

### Analysis of CAT and GPx enzyme activities

**Figure 4:**
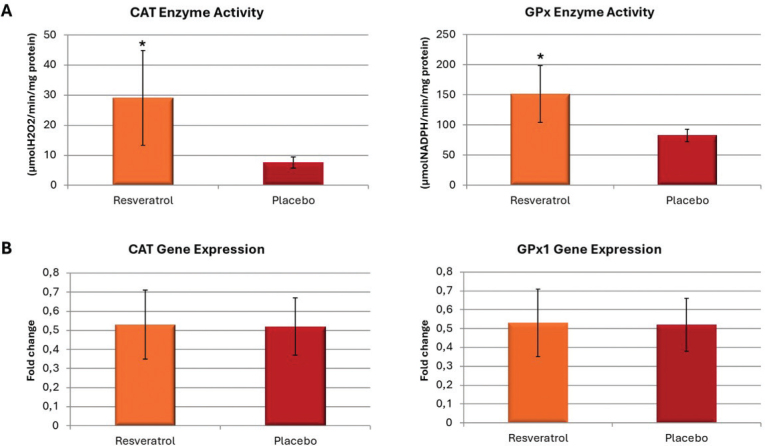
(A) Means ± SD of catalase (CAT) and glutathione peroxidase (GPx) gingival levels. *Represents significant difference between groups (Student’s *t* test, *p* < 0.05). (B) Fold changes of CAT and GPx1 gingival gene expression levels (Student’s t test, *p* > 0.05).

The enzyme activity and gene expression levels in gingival tissue samples from periodontitis-induced mandibular molars were compared between the RESV and PLAC groups. The mean CAT activity in the RESV (29.11 ± 15.72) group showed a 3.8-fold increase compared to PLAC (7.59 ± 1.93) (*p* < 0.001). Mean total GPx activity in the RESV group (151.23 ± 47.13) was significantly higher compared to the PLAC (82.65 ± 10.37) group (*p* < 0.001) (Figure 4).

### Expression analysis of CAT and GPx1 genes

*CAT* gene expression levels showed no significant difference between the RESV (0.53 ± 0.18) and PLAC (0.52 ± 0.15) groups (p>0.05). Similarly, *GPx1* mRNA levels, did not differ significantly between the RESV (0.53 ± 0.18) and PLAC (0.52 ± 0.14) groups (p>0.05) (Figure 4).

## Discussion

The present study was designed to explore the impact of resveratrol on periodontal tissue damage in a rat experimental periodontitis model. This was achieved by assessing alveolar bone loss and the gingival tissue mRNA levels and enzyme activities of CAT and GPx1 30 days after application of daily 10 mg/kg resveratrol via gavage.

This dosage was selected because it has been consistently employed in previous studies with similar experimental designs [21, 28, 32], as also highlighted in the meta-analysis by Andrade et al. [44] and found to be effective in reducing alveolar bone loss (ABL). Higher doses have also been reported in the literature; for example, Chin et al. [45] employed 25 mg/kg in a rat periodontitis model but observed no significant improvement, possibly due to the short 7-day treatment period. These findings suggest that both dose and treatment duration may influence the therapeutic effects of resveratrol. With respect to toxicity, no side effects were reported in the previous studies using similar experimental design [44] and several investigations in rats have further demonstrated the safety of resveratrol [46–48].

The effects of resveratrol on bone metabolism have been extensively investigated in previous studies. A systematic review and meta-analysis specifically evaluated whether resveratrol administration could control the progression of induced periodontal disease in preclinical models. The results demonstrated that resveratrol treatment significantly reduced alveolar bone loss, supporting its potential as a protective agent in periodontitis [44].

In line with these findings, resveratrol has been shown to positively influence bone metabolism through its dual role as a selective estrogen receptor modulator (SERM) and a mixed agonist/antagonist, as well as through its anti-inflammatory and antioxidant effects. It promotes osteoblast differentiation and bone formation by modulating various signaling pathways. Moreover, RESV reduces bone resorption by inhibiting osteoclast activity, primarily through the RANKL/RANK/OPG pathway**,** which blocks osteoclastogenesis [16].

Consistent with this body of evidence, our findings indicate that systemic RESV administration results in reduced alveolar bone loss compared to placebo. This observation aligns with previous studies demonstrating decreased bone loss associated with resveratrol use in experimental periodontitis models in rats [14–16, 20, 21, 31, 32].

Alveolar bone loss in periodontitis is primarily driven by the host’s inflammatory response to microbial biofilms, which is closely associated with oxidative stress. Reactive oxygen species contribute not only to direct oxidative damage of periodontal tissues but also to the activation of signaling pathways that enhance osteoclastogenesis and suppress osteoblast function [49, 50]. Resveratrol, through its antioxidant and anti-inflammatory properties, may mitigate these processes by reducing ROS production, modulating cytokine release, and regulating the RANKL/RANK/OPG axis [51]. By attenuating oxidative stress–mediated bone resorption and supporting bone formation, resveratrol could exert a dual protective role in periodontal disease progression [44].

Oxidative stress has been widely recognized as a pivotal factor in the pathogenesis of periodontitis, primarily due to the imbalance between ROS production and the antioxidant defense system [8]. Reactive oxygen species are continuously generated as a part of normal cellular metabolism and are neutralized by enzymatic antioxidant systems within the cell [52]. Overproduction of ROS, however, reduces the activities of enzymatic antioxidants, such as CAT and GPx which participate in cellular defense. When the activity levels of CAT and GPx (alongside SOD) are diminished, it can lead to an overwhelming accumulation of ROS, causing disruption of cellular membrane structures and interfering with normal cell functions [53, 54]. The main enzymatic antioxidants that directly combat free radicals include CAT, which decomposes hydrogen peroxide (H₂O₂) into water and oxygen, and GPx, which also reduces H₂O₂ to water, thereby preventing the formation of the highly reactive hydroxyl radical (•OH) [55]. The interplay between the production of H₂O₂, due to the dismutation of O₂ facilitated by SOD, and its subsequent removal by CAT and GPx creates a finely tuned balance [56, 57].

The enhancement of cellular antioxidant systems helps to minimize oxidative stress and protect tissues from damage caused by ROS [58, 59]. Several studies have documented the application of different antioxidants as a strategy to counteract the harmful consequences of oxidative stress on periodontal tissues [60–62]. In the literature, studies evaluating the use of antioxidants and their impact on catalase levels in experimental periodontitis models have reported notable findings. For instance, Demkovych et al. demonstrated that antioxidant administration resulted in high levels of catalase activity in blood plasma, stabilizing the free-radical oxidation [63]. Similarly, Kocaman et al. observed an increase in CAT enzyme activity in their study investigating the protective effects of the antioxidant properties of crocin against oxidative damage in experimental periodontitis, with catalase activity notably elevated compared to the control group [64].

Parallel with previous findings in the literature, the current study demonstrated that CAT activity in the RESV group increased by 3.8-fold compared to the PLAC group. Considering this result together with the lower levels of bone destruction observed in the same group, it can be interpreted that resveratrol promotes the cellular antioxidant defense and protects tissues by scavenging free radicals. With regards to CAT gene expression levels, no significant differences were found between PLAC and RESV groups. These results suggest that CAT activity is controlled at the protein level, not the mRNA level. The significant increase in CAT activities in RESV group can be explained by post-translational modifications due to an increased phosphorylation state. To date, there are no publications that evaluated the effect of resveratrol on CAT gene expression and enzymatic activity in periodontitis; hence, this finding is yet to be discussed.

GPxs remove H₂O₂ by oxidizing glutathione (GSH) to its oxidized form (GSSH). GPx1, a cytosolic and mitochondrial enzyme, reduces fatty acid hydroperoxides and H₂O₂ at the expense of glutathione and is present in most tissues [44]. Various previous studies investigating the effects of different antioxidants in experimental periodontitis have also assessed GPx activity. Paksoy et al. observed an increase in GPx enzyme activity in their experimental periodontitis model with antioxidant treatment [65]. Similarly, Salnykov et al. reported elevated GPx-4 levels in the group treated with the antioxidant Selenase [66]. In line with these findings, our study found that total GPx activity was increased in the RESV group compared to the PLAC group, although no difference was observed in GPx gene expression levels between the groups. Increments in total GPx activity may be correlated to post-translational modifications of GPx1 and/or altered gene expression levels of other peroxidases, particularly GPx4[67]. GPx may act as a buffer zone to control the overall level of ROS, and CAT may have a more predominant role in removal of high H_2_O_2_ that reaches to different cellular compartments during oxidative stress. In concordance, the increase in CAT levels were more pronounced.

We interpret these observations as indicating there is no direct link between mRNA expression levels and enzyme activity for both CAT and GPx1. As pointed out, this gap may be due to post-transcriptional and post-translational regulation which can significantly influence how proteins are translated and how enzymes function. Additionally, the use of a single time-point experimental design could contribute to the observed differences between gene expression and enzymatic activity. Similar inconsistencies have been observed in other models of inflammation and oxidative stress, where mRNA levels do not always correlate with the amount of functional protein, due to factors such as mRNA stability, translation efficiency, and enzyme turnover [68–71].

This study is the first to evaluate the effect of resveratrol administration on gingival tissue mRNA levels and enzyme activities of CAT and GPx1 in a rat experimental periodontitis model simultaneously. The results of this study may provide promising insights into the potential of resveratrol to decrease bone loss in experimental periodontitis as well as its impact on antioxidant mechanisms through systemic administration. Despite the valuable findings presented in this study, several limitations must be acknowledged. First, all measurements were conducted at a single time point (day 30), which restricts our ability to assess the temporal dynamics of resveratrol’s effects on antioxidant enzyme expression and activity. Future studies employing multi-time-point experimental designs are recommended to better elucidate the time-dependent modulation of antioxidant responses by resveratrol. Second, this study focused on the relative differences among ligated groups to assess the therapeutic potential of resveratrol within a disease model and therefore did not include non-ligated control groups. This limits the ability to evaluate baseline antioxidant levels and increases the risk of bias in interpreting resveratrol’s modulatory effects. Third, oxidative stress was evaluated using only two enzymatic markers (CAT and GPx1), which may not comprehensively reflect the broader oxidative status. Future research should include additional markers such as superoxide dismutase (SOD), malondialdehyde (MDA), 8-hydroxy-2’-deoxyguanosine (8-OHdG), and total antioxidant capacity (TAC) to provide a more comprehensive assessment. Lastly, the reliance on an animal model for this research restricts the direct extrapolation of the findings to human clinical settings. Therefore, while our results provide promising preclinical evidence regarding the protective effects of resveratrol on periodontal tissues, further validation through well-controlled clinical studies to assess efficacy, optimal dosing, safety, and long-term outcomes is necessary.

## Conclusion

Within the limitations of this study, it can be concluded that resveratrol has a modulatory effect on antioxidant defense mechanisms in experimental periodontitis, reflected by changes in enzymatic activities and gene expression of key antioxidants such as catalase and GPx. These findings highlight the potential therapeutic benefits of resveratrol in mitigating oxidative stress-related periodontal tissue damage such as catalase and glutathione peroxidase.

## Data Availability

The data that support the findings of this study are available from the corresponding author upon reasonable request.
